# Excimer laser coronary angioplasty: a mini-narrative review of clinical outcomes

**DOI:** 10.1186/s43044-024-00561-8

**Published:** 2024-09-16

**Authors:** Gbolahan Olatunji, Emmanuel Kokori, John Aboje, Saad Mohammed, Olamide Asifat, David Timilehin Isarinade, Ismaila Ajayi Yusuf, David B. Olawade, Nicholas Aderinto

**Affiliations:** 1 Department of Medicine and Surgery, University of Ilorin, Ilorin, Nigeria; 2 College of Health Sciences, Benue State University, Benue, Nigeria; 3 Al - Kindy College of Medicine, University of Baghdad, Baghdad, Iraq; 4 Department of Medicine and Surgery, Obafemi Awolowo University Teaching Hospital, Ife, Nigeria; 5 Department of Allied and Public Health, School of Health, Sport and Bioscience, University of East London, London, UK; 6 Department of Medicine and Surgery, Ladoke Akintola University Teaching Hospital, Ogbomoso, Nigeria

**Keywords:** Excimer laser coronary angioplasty, Percutaneous coronary intervention, Clinical outcomes, Safety

## Abstract

**Background:**

Excimer laser coronary angioplasty (ELCA) has evolved as a pivotal element in percutaneous coronary intervention (PCI), significantly influencing procedural efficacy and safety. This mini-narrative review explores ELCA's applications, focusing on its efficacy and clinical outcomes.

**Body:**

A search of major databases identified studies from ELCA's inception. Inclusion criteria encompassed diverse study designs exploring ELCA in coronary interventions, with rigorous data extraction ensuring accuracy and completeness. A narrative synthesis presented key findings across studies. ELCA demonstrated promising outcomes compared to traditional PCI and stent placement. Reduced reperfusion time, enhanced microcirculation, and lower postoperative major adverse cardiac events (MACE) rates highlighted its efficacy. Improved vascular and lumen dynamics, plaque modification, and successful treatment of complex lesions showcased its versatility. Quality of life enhancements positively impacted long-term recovery, particularly in acute coronary syndrome (ACS) cases. ELCA's success in challenging scenarios and its role in refining in-stent restenosis (ISR) treatment indicated broader applications. Despite limitations in some studies, ELCA presented a favorable safety profile.

**Conclusion:**

The review underscores ELCA's dynamic role in coronary interventions, offering a promising tool for enhancing procedural outcomes. Clinical implications include improved reperfusion, adaptability in complex lesions, and potential long-term benefits for ACS patients. While integration into routine practice requires careful consideration, ELCA's positive outcomes encourage further exploration and innovation in interventional cardiology.

## Background

Coronary artery disease (CAD) remains a global health crisis [1]. Percutaneous coronary intervention (PCI), also known as angioplasty, has emerged as a cornerstone treatment for this condition [2]. This minimally invasive procedure involves navigating a catheter with a tiny balloon to the blocked artery and inflating it to restore blood flow. Despite its effectiveness, conventional balloon angioplasty with stenting faces limitations. Certain complex lesions, such as heavily calcified plaques, in-stent restenosis (re-narrowing within a previously placed stent), and chronic total occlusions (complete blockage), can pose challenges for balloon dilatation alone [2]. In these scenarios, excimer laser coronary angioplasty (ELCA) offers a valuable tool within the PCI armamentarium.

ELCA has become an integral component of percutaneous coronary intervention PCI, significantly influencing both the efficacy and safety of the procedure [1]. As a complementary method, ELCA is employed alongside conventional PCI, crucial in enhancing outcomes in suitable cases [1, 2]. Originating to improve revascularisation results for patients with coronary artery disease, ELCA seamlessly integrates into the nonsurgical angioplasty process [3]. This innovative technique involves using an inflatable balloon-tipped catheter to address coronary artery constriction or blockage resulting from underlying atherosclerosis [3, 4].

In response to the limitations posed by earlier laser technologies, ELCA has evolved into a dynamic tool within nonsurgical angioplasty procedures [5, 6]. Its application has demonstrated particular promise in treating challenging lesions resistant to conventional methods [7, 8]. Lasers, including excimer lasers, consist of an excitation source (pump) and an optical resonator with mirrors [9]. The active medium (solid, liquid, or gas) determines the laser's emitted wavelength, with the excitation source energizing atoms in the active medium [10]. Laser function is governed by stimulated emission of radiation, producing intense, monochromatic, and coherent light [11, 12].

In interventional cardiology, lasers operate in pulses, delivering high-intensity light through fiber optics [13]. Excimer lasers, utilizing xenon gas and hydrogen chloride, emit UV light (308 nm) with minimal tissue absorption, thus reducing collateral damage [14]. The wavelength determines penetration depth, with UV lasers exhibiting less depth and heat, making them advantageous for clinical applications [15]. Over the past 25 years, ELCA has played a pivotal role in percutaneous interventions for coronary artery stenoses, garnering renewed interest in treating various complex lesion subsets [16]. Utilizing a pulsed ultraviolet laser catheter, ELCA has proven effective with minimal thermal damage, as demonstrated in its initial application for atherosclerotic tissue vaporization in 1985 [17]. Recent reports highlight its resurgence in addressing complex coronary diseases, including in-stent restenosis (ISR), debulking Saphenous Vein Graft lesions, interventions for Chronic Total Occlusion, and treatment of thrombotic lesions [18–20].

With ongoing procedural refinements, advancements in laser technology, and expanding clinical applications, ELCA stands as a dynamic and promising tool in the evolving landscape of interventional cardiology. Its renewed success in managing complex coronary diseases suggests exciting prospects for further innovation and contributions to improved patient outcomes. This review aims to comprehensively evaluate the applications of ELCA, with a specific focus on assessing its efficacy, safety profile, and clinical outcomes.

## Methods

### Literature search strategy

We initiated our review by systematically searching major electronic databases, including PubMed, MEDLINE, Embase, and the Cochrane Library. Employing a comprehensive set of keywords such as "Excimer Laser," "Coronary Angioplasty," "ELCA Efficacy," "ELCA Safety," and "Clinical Outcomes of ELCA," we aimed to capture a diverse array of studies dating from the inception of ELCA technology to the December, 2023.

### Inclusion and exclusion criteria

Our criteria for inclusion involved studies that looked into ELCA in coronary interventions, encompassing diverse research designs, such as randomized controlled trials (RCTs), observational studies, case series, and reviews. Exclusions were made for studies lacking primary outcome measures pertinent to ELCA efficacy, safety, or clinical outcomes, reviews, conference abstracts, as well as those offering insufficient data or presenting inadequate reporting and studies published more than a decade ago.

### Data extraction

Systematic and thorough data extraction was performed from the selected studies. This involved gathering details on study design, sample size, patient demographics, procedural intricacies, and primary outcomes encompassing efficacy, safety, and clinical outcomes. Rigorous cross-verification ensured the extracted data's accuracy, consistency, and completeness.

### Data synthesis and analysis

A narrative synthesis was undertaken to group key findings, presenting a cohesive summary of outcomes and trends across studies.

## Current evidence for ELCA

The ELCA has demonstrated promising results compared to alternative treatment modalities, including traditional coronary angioplasty and stent placement. Multiple studies have reported positive outcomes, offering valuable insights into the potential advantages of this therapeutic approach (see Table 1).

**Table 1:** Study characteristics

Author & year	Study design	Sample population	Objectives	Positive outcomes	Negative outcomes	Summary of Key findings
Arai et al. [27]	The study was retrospective and analyzed 113 consecutive patients with acute coronary syndrome (ACS) who underwent percutaneous coronary intervention (PCI) with either excimer laser coronary angioplasty (ELCA) or manual thrombus aspiration therapy (TA). The patients had a Thrombolysis in Myocardial Infarction (TIMI) flow grade of 0 within 24 h of onset. The data was collected between March 2011 and March 2020	A total of 113 ACS patients (48 in the ELCA group and 50 in the TA group)	The study aimed to compare the outcomes of ELCA and TA in ACS patients with a TIMI grade 0. The primary objectives included assessing door-to-reperfusion time, myocardial blush grade (MBG) before and after treatment, and in-hospital major adverse cardiac events (MACE)	The ELCA group had a significantly shorter door-to-reperfusion time compared to the TA group Post-treatment MBG scores were significantly higher in the ELCA group In-hospital major adverse cardiac events (MACE) were significantly fewer in the ELCA group compared to the TA group	The peak creatine kinase (CK) levels were not significantly different between the ELCA and TA groups	The study found that ELCA for ACS patients with TIMI grade 0 was associated with shorter reperfusion times, improved MBG scores, and reduced in-hospital MACE compared to TA. The use of ELCA appears to be a beneficial revascularization technique for these patients
Shimojo et al. 2023	This study is a retrospective analysis of patients with ST-segment elevation myocardial infarction (STEMI) who underwent primary percutaneous coronary intervention (PCI) using either excimer laser coronary angioplasty (ELCA) or manual aspiration thrombectomy conducted between September 2016 and December 2020	143 consecutive patients with STEMI, with 63 patients treated with ELCA and 80 patients treated with manual aspiration thrombectomy	To evaluate and compare the impact of ELCA and manual aspiration thrombectomy on myocardial salvage and LV systolic/diastolic function in patients with STEMI	ELCA suppressed myocardial enzyme elevations, leading to lower peak creatine kinase-myocardial band (CK-MB) levels Significant improvement in LV ejection fraction (LVEF) was observed in the ELCA group compared to the manual aspiration thrombectomy group There were no in-hospital major adverse cardio-cerebral events (MACCEs) in the ELCA group	No significant difference in myocardial salvage was observed between the ELCA and aspiration groups There was no significant improvement in LV diastolic function in either group	ELCA was found to be effective in suppressing myocardial enzyme elevations, potentially improving systolic function (LVEF), and reducing procedural complications compared to manual aspiration thrombectomy in patients with STEMI. However, no significant difference in myocardial salvage or LV diastolic function was observed between the two treatment methods. The study suggests that ELCA could be a feasible option for primary PCI in patients with STEMI
Sasaki et al. [25]	The study used a cross-sectional design and employed integrated backscatter-intravascular ultrasound (IB-IVUS) to assess coronary plaque composition before and after excimer laser coronary angioplasty (ELCA). The study was conducted between August 2018 and March 2020	51 patients with coronary artery disease underwent percutaneous coronary intervention (PCI) and ELCA	The primary objective was to investigate the impact of ELCA on coronary plaque composition using IB-IVUS. The study aimed to evaluate whether ELCA modified the plaque composition in addition to debulking it	The study found that after ELCA, there was an increase in the minimum lumen diameter, lumen volume, and vessel volume. Additionally, there was a decrease in the 'lipid' component of the plaque and an increase in the 'fibrous' component as observed through IB-IVUS	The study did not report any significant negative outcomes	ELCA led to lumen enlargement and vessel expansion, but it did not significantly decrease plaque volume. The most notable finding was the modification of coronary plaque composition, with a decrease in lipid-rich areas and an increase in fibrous areas after ELCA, as observed through IB-IVUS. This suggests that ELCA contributes to the alteration of coronary plaque composition in addition to debulking. Further research is needed to understand the clinical implications of these findings
Shibata etal. 2022	A retrospective analysis of 316 consecutive patients who underwent primary percutaneous coronary intervention (p-PCI) after their first ST-elevation myocardial infarction (STEMI) in a single institute between September 2014 and April 2017. The primary focus was to evaluate the impact of excimer laser coronary atherectomy (ELCA) on myocardial salvage using nuclear scintigraphy. The data was collected between September 2014 and April 2017, spanning approximately 2.5 years	72 patients with STEMI, initial thrombolysis in myocardial infarction (TIMI) flow-0/1, and onset-to-balloon time (OBT) of less than 6 h were included in the study. Among them, 32 patients received ELCA, while 40 did not	The study assessed the impact of ELCA on myocardial salvage in patients with STEMI. Myocardial salvage was evaluated using a 17-segment model with a 5-point scoring system	The study suggested that ELCA might improve myocardial salvage in patients with STEMI, particularly when final TIMI-3 flow was achieved	The study did not report any significant negative outcomes related to the use of ELCA. There were no in-hospital major adverse cardio-cerebral events (MACCE) in the ELCA group	The study found that ELCA has the potential to improve myocardial salvage in patients with STEMI, especially in cases where final TIMI-3 flow is achieved. The use of ELCA also allowed for the direct implantation of shorter stents without an increased risk of peri-procedural complications. The authors suggested that ELCA may be a safe and effective option for improving myocardial salvage in these patients
Harima et al. [24]	Non-randomized controlled trial Intervention: A novel stent-less revascularization strategy using a combination of excimer laser coronary angioplasty (ELCA) and drug-coated balloon (DCB) for patients with acute coronary syndrome (ACS). The study was conducted from February 2014 to January 2016	249 ACS patients were initially considered. 86 patients were allocated to the DCB with ELCA arm, while 94 patients were allocated to the Stent without ELCA arm. 26 patients in the DCB with ELCA arm eventually underwent unplanned stenting. 168 patients received the scheduled procedure successfully	To test the feasibility and validity of a novel stent-less revascularization strategy for ACS patients using a combination of ELCA and DCB. To assess the outcomes of major cardiac adverse events (MACEs), target vessel revascularization (TVR), and angiographic results	MACE rate did not significantly differ between the groups (10%, 4.3%, and 3.5%). No myocardial infarctions occurred during the follow-up period. TVR was more common in the DCB with ELCA group (15%) compared to the Stent with ELCA group (0%). The angiographic outcome was favorable in patients who underwent stenting following ELCA	A higher incidence of restenosis was observed in the DCB with ELCA group, particularly at 6-month follow-up (25.7%). Approximately one-third of patients scheduled for DCB and ELCA eventually underwent unplanned stenting	The stent-less revascularization strategy using DCB and ELCA was associated with a higher occurrence of restenosis in ACS patients, particularly at 6-month follow-up. Although the study did not show significant differences in MACE rates across treatment groups, TVR was more common in the DCB with ELCA group. Unexpectedly, patients who received stents following ELCA had favorable angiographic outcomes compared to those receiving conventional stent-based angioplasty
Hirose et al. [26]	Retrospective, single-center clinical trial The study was conducted between April 2010 and July 2014	The study included 23 patients with in-stent restenosis (ISR) lesions Patients received either excimer laser coronary angioplasty (ELCA) followed by scoring balloon dilatation or scoring balloon dilatation alone	The study aimed to evaluate the clinical safety and 6-month efficacy of ELCA followed by scoring balloon dilatation for the treatment of ISR	Procedural success was achieved in all patients The late luminal loss was significantly lower in the ELCA group compared to the non-ELCA group ELCA was associated with a relatively low recurrence of restenosis compared to scoring balloon dilatation alone	The incidence of target lesion revascularization (TLR) was not significantly different between the ELCA and non-ELCA groups The study had some limitations, including a small sample size and the absence of randomization	The study found that ELCA is a safe and feasible technique for treating ISR lesions ELCA, when used before scoring balloon dilatation, was associated with better outcomes, including reduced late luminal loss, compared to scoring balloon dilatation alone ELCA may help decrease the pressure needed for scoring balloon expansion and improve long-term results in the treatment of ISR lesions
Ambrosini et al. [21]	The study utilized an innovative xenon–chlorine (excimer) pulsed laser catheter (ELCA X80) to treat complex coronary lesions, including calcified stenosis, chronic total occlusions, and non-compliant plaques	Eighty patients with 100 coronary lesions participated in the study The target lesions included calcified stenosis, chronic total occlusions, and balloon-resistant lesions The patients were referred from four different medical centers and excimer laser coronary angioplasty was performed on 96 lesions	The main objective was to examine the acute outcome of using the ELCA X80 excimer laser catheter on patients with complex coronary lesions The study aimed to assess the safety and effectiveness of this approach and evaluate if higher laser energy levels improved the success rates without increasing complications	Laser success was achieved in 93.7% of the lesions Procedural success was reached in 91.7% of cases Clinical success was obtained in 90.6% of cases No major complications, such as perforation, dissection, spasm, no-reflow phenomenon, or acute vessel closure, were reported Higher laser parameters were used successfully for 49 resistant lesions	The study suggested that the ELCA X80 excimer laser catheter is a simple, safe, and effective device for treating complex coronary lesions, including calcified stenosis, chronic total occlusions, and non-compliant plaques Higher laser energy levels delivered by this catheter improved device performance without increasing complications, leading to high success rates for laser-facilitated coronary angioplasty	

### Reduced reperfusion time

In the 2023 Ambrosini et al. 2015 study, ELCA demonstrated a shorter door-to-balloon time than Thrombus Aspiration (TA) [21]. The ELCA group exhibited an enhanced Myocardial Blush Grade (MBG) compared to the TA group. ELCA significantly improved postoperative MBG scores compared to TA, indicating enhanced microcirculation. Moreover, the ELCA group demonstrated a significantly lower postoperative Major Adverse Cardiac Events (MACE) rate than the TA group. Similarly, Shimojo et al. (2023) reported promising outcomes with ELCA, suggesting a potential reduction in infarction size and an improvement in Left Ventricular (LV) function. Improved LV ejection fraction (EF), peak emptying rate (PER), and LV systolic function were evident in the ELCA group [22]. ELCA not only led to improved postoperative MBG scores, signifying enhanced microcirculation compared to TA, but also demonstrated improvements in LV systolic function, particularly in ejection fraction and peak emptying rate. While ELCA showed positive effects on blood flow, its impact on plaque modification appeared promising despite limitations in reducing plaque volume. Shibata et al. 2022 showed that ELCA enhanced myocardial salvage in ST-segment elevation myocardial infarction (STEMI) patients treated within 6 h after onset and with initial TIMI flow-0/1 [23]. Harima et al. (2018) noted that the ELCA group exhibited a lower late luminal loss at follow-up than the non-ELCA group, indicating better outcomes in maintaining improved blood flow [24]. This improvement was attributed to the healing process of reducing neointimal plaque and modifying the neointima after scoring balloon expansion. ELCA, when performed before scoring balloon dilatation, showed a lower late luminal loss, suggesting a potential enhancement in blood flow and reduced neointimal tissue regrowth.

### Vascular and lumen volume enhancement

According to Sasaki et al.'s 2022 study, ELCA increased vessel and lumen volume while plaque volume remained constant [25]. Integrated backscatter-IVUS (IB-IVUS) analysis revealed a shift in plaque composition, indicating a decrease in lipid plaque and an increase in fibrous plaque following ELCA. Shibata et al. 2022 showed that ELCA facilitated the implantation of shorter stents, reducing the need for longer stents and minimizing the number of balloons used during the procedure [23]. Harima et al.'s 2018 study emphasized ELCA's success in treating diffuse lesions, chronic total occlusions, and in-stent restenosis [24]. Additionally, Drug-coated balloon (DCB) showed efficacy in releasing antiproliferative agents into vessel walls, inhibiting intimal hyperplasia. In cases of acute coronary syndrome (ACS), ELCA alone or DCB following rotational atherectomy successfully treated patients without the need for stenting.

Hirose et al. 2016 reported positive outcomes for ELCA in treating in-stent restenosis (ISR) [26]. Before scoring balloon dilatation, ELCA resulted in relatively low recurrent restenosis compared to scoring balloon dilatation alone, establishing ELCA as a safe and feasible technique for ISR treatment. Arai et al.'s 2023 findings aligned with previous studies, indicating procedural success for all patients, significantly lower late luminal loss in the ELCA group compared to the non-ELCA group, and relatively lower recurrent restenosis compared to scoring balloon dilatation alone [27]. ELCA demonstrated efficiency in plaque removal and thrombus vaporization, potentially stabilizing thrombus within culprit lesions. It appeared effective in reducing myocardial enzyme elevations, indicative of reduced plaque burden and a smaller infarction size. Notably, Sasaki et al., 2022 reported high success rates in achieving lumen expansion, particularly in ST-segment elevation myocardial infarction (STEMI) cases [23]. Shibata et al. 2022 suggested that ELCA usage resulted in shorter stent lengths and reduced balloon usage, indicating improved plaque and thrombus removal, potentially leading to direct stenting and less myocardial damage [23]. Similarly, Harima et al., 2018 reported the excimer laser's success in treating various lesion types, including diffuse lesions, chronic total occlusions, and in-stent restenosis [24]. In Hirose et al.'s 2016 study, ELCA effectively reduced neointimal plaque area, though the exact mechanism behind this advantage remained unclear [26].

### Quality of life improvements

The shortened reperfusion times achieved with ELCA contribute significantly to better outcomes and improved quality of life for patients experiencing Acute Coronary Syndrome (ACS) [23, 24]. The ELCA group exhibited promising outcomes, including quicker reperfusion and improved blood flow. It reduced adverse events compared to Thrombus Aspiration (TA), suggesting its potential as a favorable intervention for ACS. In the study by Shimojo et al. (2023), improvements in Left Ventricular (LV) function and the potential reduction in infarction size with ELCA were highlighted, contributing to a better quality of life for patients recovering from ST-segment elevation myocardial infarction (STEMI) [22]. Additionally, findings from the study conducted by Sasaki et al. (2022) suggest that ELCA can modify plaque composition, leading to improved blood flow and potentially better patient outcomes [25].

The study by Shibata et al. (2022) indicated that ELCA offers advantages in myocardial salvage, potentially reducing infarction size and preserving cardiac function, thereby positively impacting patients' long-term quality of life. [23] ELCA showed promise in improving myocardial salvage in STEMI patients undergoing percutaneous coronary intervention (PCI), suggesting its potential as a beneficial strategy for this specific patient population. Moreover, the study by Hirose et al. (2016) suggests that using ELCA as a pre-treatment strategy before scoring balloon dilatation is associated with better outcomes in In-Stent Restenosis (ISR) treatment [26]. This is primarily attributed to ELCA's impact on reducing neointimal plaque, facilitating better long-term results, and maintaining improved blood flow, collectively enhancing the quality of life for patients undergoing this particular intervention.

### Study limitations

The study conducted by Arai et al. [27] faced limitations due to its retrospective, single-center design with a relatively small sample size and a mixed population, including non-ST segment elevation myocardial infarction and unstable angina pectoris, exceeding recommended reperfusion times. The peak creatine kinase (CK) levels did not show significant differences between the ELCA and Thrombus Aspiration (TA) groups. Despite the benefits of ELCA, there was no demonstrated advantage in other parameters reflecting myocardial viability or Left Ventricular (LV) diastolic function. No significant difference in myocardial salvage was observed between the ELCA and aspiration groups, and there was no significant improvement in LV diastolic function in either group.

Harima et al. [24] revealed that unplanned stenting was more common than expected in patients initially scheduled for Drug-coated Balloon (DCB) with ELCA. The combined strategy of DCB and ELCA did not yield a more gratifying outcome compared to traditional stent-based interventions. Shibata et al. 2022 also reported a limited effect on Final Thrombolysis in Myocardial Infarction-3 (TIMI-3) flow and slow-flow prevention with ELCA [23]. ELCA did not demonstrate a significant advantage in achieving final TIMI-3 flow or preventing slow-flow phenomena compared to conventional Percutaneous Coronary Intervention (PCI), possibly due to the reduced use of aspiration therapy and distal protection devices in the ELCA group.

In terms of morbidity and mortality, the Shibata et al. 2022 study suggested that ELCA could be beneficial for myocardial salvage in early ST-segment elevation myocardial infarction (STEMI) cases but did not show clear advantages in achieving final TIMI-3 flow or preventing slow-flow complications compared to conventional PCI [23]. Harima et al., 2018 reported that about one-third of patients initially intended for DCB with ELCA underwent unplanned stenting, potentially affecting procedural safety [24]. The study reported similar frequencies of major adverse cardiac events (MACEs) across treatment groups but varied rates of target vessel revascularisation (TVR). No myocardial infarctions were reported during the follow-up period, and the overall mortality rate was not mentioned. Bleeding events requiring transfusion or surgical repair occurred in a small percentage (3.3%) of patients in the DCB with ELCA group during follow-up. Similarly, according to Hirose et al. [26], no procedure-associated adverse events such as myocardial infarction, emergency bypass surgery, or in-hospital deaths were recorded during the study.

### Future directions and recommendation

The examination of ELCA in this review established its effectiveness compared to alternative methods like traditional coronary angioplasty and stent placement.

The demonstrated reduction in door-to-balloon time and improved microcirculation associated with ELCA, as evidenced by elevated MBG and reduced MACE rates, suggests a tangible benefit for patients experiencing acute coronary events. The swift reperfusion achieved through ELCA has direct implications for minimizing myocardial damage and improving overall patient outcomes. The positive impact of ELCA on vascular and lumen dynamics, including increased vessel and lumen volume and favorable shifts in plaque composition, opens avenues for more refined and patient-specific interventions. This enhancement in procedural adaptability, particularly in optimizing stent placement and addressing diverse lesion complexities, has practical implications for tailoring interventions to individual patient needs.

The shortened reperfusion times achieved with ELCA contribute to immediate clinical benefits and potential improvements in long-term quality of life for patients recovering from ACS. The reduction in infarction size, modifications in plaque composition, and improvements in LV function collectively suggest a positive impact on patients' overall well-being and functional recovery post-intervention. The findings indicating ELCA's efficacy in reducing late luminal loss, recurrent restenosis, and improving blood flow have implications for reducing procedural complications. This may result in fewer adverse events, including target vessel revascularisation (TVR) and major adverse cardiac events (MACE), contributing to improved procedural safety and long-term stability.

ELCA's success in treating challenging lesions such as diffuse lesions, chronic total occlusions, and in-stent restenosis suggests its utility in scenarios where traditional interventions may face limitations. This expands the armamentarium of interventional cardiologists, offering a potential solution for cases historically associated with increased procedural complexity. ELCA's positive outcomes in treating ISR, as indicated by reduced neointimal plaque area and relatively lower recurrent restenosis rates, suggest a role in refining ISR treatment strategies. The potential to stabilize thrombus within culprit lesions and minimize myocardial enzyme elevations contributes to the safety and feasibility of ELCA in this specific context.

ELCA's positive impact on myocardial salvage, particularly in early ST-segment elevation myocardial infarction (STEMI) cases, positions it as a consideration in the acute phase of ACS. The potential benefits of preserving cardiac function and reducing infarction size may influence decision-making in the emergent setting. The overall positive outcomes presented in this review prompt consideration for integrating ELCA into routine clinical practice. However, the learning curve associated with ELCA and the need for careful patient selection should be acknowledged. Institutions may need to evaluate the feasibility of incorporating ELCA into their standard protocols, considering its potential benefits and challenges.

### Limitations and strengths

The review presents robust arguments that enhance its reliability and contribute to a better understanding of ELCA. Nevertheless, certain limitations need to be acknowledged. The review exclusively focuses on studies between 2015 and 2023, potentially excluding significant research conducted before or after this timeframe. Additionally, by restricting the scope to papers written in English, there is a possibility of overlooking valuable studies published in other languages, thereby introducing a language bias.

## Conclusions

This review reveals ELCA's transformative potential in PCI. The evidence across various studies positions ELCA as a promising therapeutic intervention, showcasing its efficacy and safety compared to traditional approaches like coronary angioplasty and stent placement. These outcomes have direct implications for minimizing myocardial damage during acute coronary events, thereby presenting a tangible clinical benefit. ELCA's positive impact on vascular and lumen dynamics, including enhanced vessel and lumen volume, underscores its versatility in addressing diverse lesion complexities, providing clinicians with a nuanced approach to tailored interventions.

Moreover, ELCA exhibits a promising role in improving the quality of life for patients recovering from ACS. The observed reduction in infarction size, plaque composition modifications, and LV function enhancements collectively contribute to a favorable long-term prognosis. ELCA's success in treating challenging scenarios, such as ISR and complex lesions, expands the repertoire of interventional cardiology, offering a potential solution where traditional methods might face limitations. Despite these promising outcomes, the review acknowledges the limitations in some studies, emphasizing the need for careful consideration and patient selection. The learning curve associated with ELCA and the potential challenges necessitate a balanced evaluation for its seamless integration into routine clinical practice.

## Data Availability

No new datasets were generated for this study. All data used are within this manuscript.

## Abbreviations

ELCA: Excimer laser coronary angioplasty
PCI: Percutaneous coronary intervention
MBG: Myocardial blush grade
MACE: Major adverse cardiac events
UV: Ultraviolet
ISR: In-Stent restenosis
STEMI: ST-segment elevation myocardial infarction
DCB: Drug-coated balloon
TIMI: Thrombolysis in myocardial infarction
LV: Left ventricular
EF: Ejection fraction
PER: Peak emptying rate
IB-IVUS: Integrated backscatter-intravascular ultrasound
ACS: Acute coronary syndrome
RCTs: Randomized controlled trials
TVR: Target vessel revascularization
MOR: Morbidity and mortality
CK: Creatine kinase

## References

[1] Mahmood UA, Hajj G. Excimer Laser Coronary Angioplasty. [Updated 2023 Jul 24]. In: StatPearls [Internet]. Treasure Island (FL): StatPearls Publishing; 2023 Jan-. Available from: https://www.ncbi.nlm.nih.gov/books/NBK563198/33085345

[2] Rawlins J, Din JN, Talwar S, O’Kane P (2016) Coronary intervention with the excimer laser: review of the technology and outcome data. Interv Cardiol (London, England). 11(1):27–32. 10.15420/icr.2016:2:2 10.15420/icr.2016:2:2 PMC5808483 29588701

[3] Chhabra L, Zain MA, Siddiqui WJ. Angioplasty. In: StatPearls. StatPearls Publishing; 2023. 29763069

[4] Grundfest WS, Litvack F, Forrester JS, Goldenberg T, Swan HJ, Morgenstern L, Fishbein M, McDermid IS, Rider DM, Pacala TJ (1985) Laser ablation of human atherosclerotic plaque without adjacent tissue injury. J Am Coll Cardiol 5(4):929–933. 10.1016/s0735-1097(85)80435-6 3838324 10.1016/s0735-1097(85)80435-6

[5] Waller BF (1989) Crackers, breakers, stretchers, drillers, scrapers, shavers, burners, welders and melters: the future treatment of atherosclerotic coronary artery disease? A clinical-morphological assessment. J Am Coll Cardiol 13:969–987 2522472 10.1016/0735-1097(89)90248-9

[6] Choy DSJ (1988) History and state-of-the-art of lasers in cardiovascular disease. Laser Med Surg News Adv. 6:34–8 10.1089/lms.1988.6.3.34

[7] Appelman YE, Piek JJ, Strikwerda S, Tijssen JG, de Feyter PJ, David GK, Serruys PW, Margolis JR, Koelemay MJ, Montauban van Swijndregt EW, Koolen JJ (1996) Randomised trial of excimer laser angioplasty versus balloon angioplasty for treatment of obstructive coronary artery disease. Lancet (London, England). 347(8994):79–84. 10.1016/s0140-6736(96)90209-3 8538345 10.1016/s0140-6736(96)90209-3

[8] Reifart N, Vandormael M, Krajcar M, Göhring S, Preusler W, Schwarz F, Störger H, Hofmann M, Klöpper J, Müller S, Haase J (1997) Randomised comparison of angioplasty of complex coronary lesions at a single center. Excimer laser, rotational atherectomy, and balloon angioplasty comparison (ERBAC) study. Circulation. 96(1):91–98. 10.1161/01.cir.96.1.91 9236422 10.1161/01.cir.96.1.91

[9] Bittl JA, Ryan TJ Jr, Keaney JF Jr, Tcheng JE, Ellis SG, Isner JM, Sanborn TA (1993) Coronary artery perforation during excimer laser coronary angioplasty. The percutaneous excimer laser coronary angioplasty registry. J Am College Cardiol 21(5):1158–1165. 10.1016/0735-1097(93)90240-2 10.1016/0735-1097(93)90240-2 8459071

[10] Topaz O (1995) Whose fault is it? Notes on “true” versus “pseudo” laser failure. Catheter Cardiovasc Diagn 36(1):1–4. 10.1002/ccd.1810360102 10.1002/ccd.1810360102 7489586

[11] Ting HH, Timimi FK, Bittl JA (1999) Excimer laser angioplasty. Contemporary concepts in cardiology. Developments in cardiovascular medicine, vol 217. Springer, Boston

[12] Litvack F, Eigler N, Margolis J, Rothbaum D, Bresnahan JF, Holmes D, Untereker W, Leon M, Kent K, Pichard A (1994) Percutaneous excimer laser coronary angioplasty: results in the first consecutive 3,000 patients. The ELCA Investigators. J Am Coll Cardiol. 23(2):323–329. 10.1016/0735-1097(94)90414-6 8294681 10.1016/0735-1097(94)90414-6

[13] Egred M, Brilakis ES (2020) Excimer laser coronary angioplasty (ELCA): fundamentals, mechanism of action, and clinical applications. J Invasive Cardiol 32(2):E27–E35 32005787 10.25270/jic/19.00325

[14] McGuff PE, Bushnell D, Soroff HS, Deterling RA Jr (1963) Studies of the surgical applications of laser (light amplification by stimulated emission of radiation). Surg Forum 14:143–145 14064488

[15] Macruz R, Martines JRM, Tupinamba HS et al (1980) Possibilidades terapeuticas do raio laser em ateromas. Arq Bras Cardiol 34:9–13 7406741

[16] Stanek F (2019) Laser angioplasty of peripheral arteries: basic principles, current clinical studies, and future directions. Diagn Interv Radiol 25(5):392–397. 10.5152/dir.2019.18515 31358488 10.5152/dir.2019.18515 PMC6727821

[17] Khan S, Schofield P (2008) Laser use in percutaneous coronary intervention. In: Norell MS, Perrins J, Meier B, Lincoff AM (eds) Essential Interventional Cardiology, 2nd edn. WB Saunders, Philadelphia, pp 219–230. 10.1016/B978-0-7020-2981-3.50021-0

[18] Litvack F, Eigler NL, Margolis JR, Grundfest WS, Rothbaum D, Linnemeier T, Hestrin LB, Tsoi D, Cook SL, Krauthamer D (1990) Percutaneous excimer laser coronary angioplasty. Am J Cardiol 66(15):1027–1032. 10.1016/0002-9149(90)90499-q 2220626 10.1016/0002-9149(90)90499-q

[19] Safian RD, Freed M, Lichtenberg A, May MA, Juran N, Grines CL, O’Neill WW (1993) Are residual stenoses after excimer laser angioplasty and coronary atherectomy due to inefficient or small devices? Comparison with balloon angioplasty. J Am Coll Cardiol 22(6):1628–1634. 10.1016/0735-1097(93)90587-q 8227830 10.1016/0735-1097(93)90587-q

[20] Niccoli G, Giubilato S, Conte M, Belloni F, Cosentino N, Marino M, Mongiardo R, Crea F (2011) Laser for complex coronary lesions: impact of excimer lasers and technical advancements. Int J Cardiol 146(2):296–299. 10.1016/j.ijcard.2010.10.092 21126783 10.1016/j.ijcard.2010.10.092

[21] Ambrosini V, Sorropago G, Laurenzano E, Golino L, Casafina A, Schiano V, Gabrielli G, Ettori F, Chizzola G, Bernardi G, Spedicato L, Armigliato P, Spampanato C, Furegato M (2015) Early outcome of high energy Laser (Excimer) facilitated coronary angioplasty ON hARD and complex calcified and balloOn-resistant coronary lesions: LEONARDO Study. Cardiovasc Revascularisation Med: Incl Mol Interv 16(3):141–146. 10.1016/j.carrev.2015.02.002 10.1016/j.carrev.2015.02.002 25708003

[22] Shimojo K, Shibata N, Takagi K et al (2023) Excimer laser coronary angioplasty versus manual aspiration thrombectomy in patients with ST-segment elevation myocardial infarction: analysed by nuclear scintigraphy. Int J Cardiovasc Imaging 39(4):831–842. 10.1007/S10554-022-02771-0 36508056 10.1007/S10554-022-02771-0

[23] Shibata N, Takagi K, Morishima I et al (2020) The impact of the excimer laser on myocardial salvage in ST-elevation acute myocardial infarction via nuclear scintigraphy. Int J Cardiovasc Imaging 36(1):161–170. 10.1007/S10554-019-01690 31451993 10.1007/S10554-019-01690

[24] Harima A, Sairaku A, Inoue I et al (2018) Real-life experience of a stent-less revascularisation strategy using a combination of excimer laser and drug-coated balloon for patients with acute coronary syndrome. J Interv Cardiol 31(3):284–292. 10.1111/JOIC.12495 29464846 10.1111/JOIC.12495

[25] Sasaki S, Nakajima K, Watanabe K et al (2022) Integrated backscatter-intravascular ultrasound and modification of plaque during excimer laser coronary angioplasty. Cardiovasc Interv Ther 37(2):354–362. 10.1007/S12928-021-00797-0 34333753 10.1007/S12928-021-00797-0 PMC8926960

[26] Hirose S, Ashikaga T, Hatano Y et al (2016) Treatment of in-stent restenosis with excimer laser coronary angioplasty: benefits over scoring balloon angioplasty alone. Lasers Med Sci 31(8):1691–1696. 10.1007/S10103-016-2039 27516177 10.1007/S10103-016-2039

[27] Arai T, Tsuchiyama T, Inagaki D, Yoshida K, Fukamizu S (2023) Benefits of excimer laser coronary angioplasty over thrombus aspiration therapy for patients with acute coronary syndrome and thrombolysis in myocardial infarction flow grade 0. Lasers Med Sci 38(1):1–7. 10.1007/S10103-022-03691-0 10.1007/S10103-022-03691-0 36542184

